# The Significance of CD20 Intensity Variance in Pediatric Patients with B-Cell Precursor Acute Lymphoblastic Leukemia

**DOI:** 10.3390/jcm12041451

**Published:** 2023-02-11

**Authors:** Andreea Nicoleta Serbanica, Delia Codruta Popa, Constantin Caruntu, Sergiu Pasca, Cristian Scheau, Ionut Vlad Serbanica, Raluca Suciu, Valeria Tica, Elisa Busescu, Luminita Nicoleta Cima, Cerasela Jardan, Mihaela Dragomir, Daniel Coriu, Andrei Colita, Anca Colita

**Affiliations:** 1 Department of Pediatrics, Carol Davila University of Medicine and Pharmacy, 050474 Bucharest, Romania; 2 Department of Pediatric Hematology and Stem Cell Transplantation, Fundeni Clinical Institute, 022328 Bucharest, Romania; 3 Department of Biochemistry, The “Carol Davila” University of Medicine and Pharmacy, 050474 Bucharest, Romania; 4 Department of Hematology, Fundeni Clinical Institute, 022328 Bucharest, Romania; 5 Department of Physiology, The “Carol Davila” University of Medicine and Pharmacy, 050474 Bucharest, Romania; 6 Department of Dermatology, ‘Prof. N.C. Paulescu’ National Institute of Diabetes, Nutrition and Metabolic Diseases, 011233 Bucharest, Romania; 7 Department of Hematology, Iuliu Haţieganu University of Medicine and Pharmacy, 400347 Cluj-Napoca, Romania; 8 Department of Endocrinology and Diabetes, Nutrition and Metabolic Diseases—“Elias” Emergency University Hospital, 011461 Bucharest, Romania; 9 Department of Endocrinology, Carol Davila University of Medicine and Pharmacy, 050474 Bucharest, Romania; 10 Department of Hematology, Carol Davila University of Medicine and Pharmacy, 420003 Bucharest, Romania; 11 Department of Hematology, Coltea Hospital, 420003 Bucharest, Romania

**Keywords:** CD20, B-cell precursor acute lymphoblastic leukemia, pediatric patients, flow cytometry, immunophenotyping, survival analysis

## Abstract

B-cell precursor acute lyphoblastic leukemia (ALL) is a common pediatric malignancy and patients may have significant benefits from monoclonal antibodies therapy with increased survival rates. Positive CD20 expression is identified in about half of these patients and its presence may serve as a prognostic factor in disease evolution. We performed a retrospective study including 114 patients diagnosed with B-ALL and evaluated the expression of CD20 through flow cytometry at diagnosis and on day 15. Additional immunophenotypic analyses as well as cytogenetic and molecular genetic analyses were also performed. We observed an increase in the mean fluorescence intensity (MFI) of CD20 between diagnosis—1.9 (1.2–3.26) and day 15: 6.17 (2.14–27.4), (*p* < 0.0001). Furthermore, we assessed that both diagnosis and day 15 CD20 MFI had an impact on RFS and OS, respectively, for cut-off values of >8.08 at diagnosis and >28.65 at day 15. In conclusion, CD20 expression appears to be a poor prognostic feature of B-ALL in pediatric patients. In this study, stratification of the outcome by the intensity of CD20 has implications concerning the allocation to rituximab-based chemotherapy and may offer new, potentially useful information for pediatric patients with B-ALL.

## 1. Introduction

B-cell precursor acute lymphoblastic leukemia (ALL) is the most common malignancy in pediatric patients [1,2,3], representing 25% of all malignancies that occur in patients before the age of 15 [4]. Although modern multidrug chemotherapy regimens have improved the 5 year survival rate by up to 90%, due to the high incidence of B-ALL, it remains a leading cause of morbidity and mortality in pediatric patients with cancer [5]. Multiple biomarkers that estimate disease evolution and survival are under investigation for a variety of hematology malignancies [6,7]. First-line chemotherapy regimens now use numerical and structural genetic abnormalities and early therapeutic response for risk stratification in order to both minimize treatment-related mortality and identify high-risk cases in need of a more aggressive approach. The new approach to ALL treatment also involves individualized treatment with monoclonal antibodies with a potentially favorable impact on the survival rate of these patients [8,9].

CD20 is a B-cell differentiation antigen with powerful expression in all normal mature B-cells, as well as most malignant mature B-cells. Nonetheless, CD20 can also be expressed, but at significantly lower levels in more immature B-cells, starting from the pro-B phase, and increasing in intensity during maturation. This is also the case in B-cell malignant equivalents, with more immature B-ALL presenting lower expression of CD20 [8]. About half of B-cell precursor ALL cases in pediatric patients have positive CD20 expression, and, as shown by some studies, up-regulation is frequent throughout classic chemotherapy regimens, especially due to corticosteroid use which plays a role in ALL blast differentiation toward mature B-cells as demonstrated by down-regulation of CD10 and CD34, and up-regulation of CD20 [8,9,10,11,12].

The main goal of this study is to analyze the impact that CD20 expression and up-regulation has on overall survival (OS) and relapse-free survival (RFS) in pediatric patients with B-ALL.

## 2. Materials and Methods

### 2.1. Study Design

We performed a retrospective study and included 114 patients diagnosed with B-ALL, between January 2017 and December 2020, in the Department of Pediatric Hematology and Oncology, Fundeni Clinical Institute, Bucharest, Romania. The diagnosis was performed using the on-site flow cytometer. Positive CD20 expression was considered when the antigen was present in more than 20% of leukemic blasts. Cytogenetic and molecular genetics analyses were also performed at diagnosis.

The patients were assigned to risk groups according to the ALL BFM trial regimens for acute lymphoblastic leukemia [13]. The main parameters for risk group stratification were: the patient age at diagnosis, initial number of leukocytes, prednisone response on day 8, and MRD on day 15 and 33. The criteria for standard risk group included: age more than 1 year old and less than 6 years old; the leukocytes number at diagnosis less than 20,000 per µL; prednisone response on day 8 less than 1000 blasts per µL and the MRD on day 15 showing less than 0.1% blasts.

The treatment was performed in according to two sequential ALL BFM trial regimens: ALL IC-BFM 2009 [14] and the AIEOP-BFM 2017 [15].

Approval for this study was obtained from the Fundeni Clinical Institute Ethics Council (Registration Number 63972/25.11.2022) and all procedures were performed in accordance with the Declaration of Helsinki. Written informed consent statements were obtained from all patients, from their parents or legal guardians, where applicable.

### 2.2. Flow Cytometry

Immunophenotypic analyses were performed on RBC-lysed whole bone and peripheral blood samples. We followed Euroflow panels for diagnosis and we validated our eight-color panel for day 15 evaluation to investigate CD20 expression in the CD19 positive population: CD58 FITC/CD13+CD33 PE/CD34 PerCP-Cy5.5/CD19 PECy7/CD10 APC/CD38 APC H7/CD20 PacB/CD45 PacO (CD58 FITC, CD13 PE, CD33 PE, CD34 PerCP-Cy5.5, CD10 APC, CD38 APC H7, BD Biosciences, Franklin Lakes, NJ, USA; CD19 PECy7, Beckman Coulter Life Sciences, Indianapolis, IN, USA; CD20 PacB, BioLegend, San Diego, CA, USA; CD45 PacO, Invitrogen, Carlsbad, CA, USA). The instrument setup was optimized daily by analyzing CS&T Beads (BD Biosciences, Franklin Lakes, NJ, USA).

Immunophenotyping at diagnosis was performed by collecting at least 200,000 cellular events, whereas for minimal residual disease (MRD) measurements at least 500,000 events were acquired from 1,000,000 stained cells, reaching 10^−4^ level of sensitivity. Cell acquisition was performed with the BD FACSLyric™ Flow Cytometry System (BD Biosciences, Franklin Lakes, NJ, USA) and data analysis was performed with the Kaluza software version 1.3 (Beckman Coulter, Miami, FL, USA). Leukemic cells were identified using a gate that included all CD19+ cells versus side scatter (SSC). MRD was defined as an accumulation of at least 50 clustered events displaying lymphoid-scattering properties and leukemia-associated immunophenotype characteristics described at diagnosis.

CD20 expression of samples was estimated by assessing the proportion of leukemic cells positive for the antigen with a cut-off of more than or equal to 20%. The threshold was set according to the upper limit of the background fluorescence of residual lymphoid cells not expressing B-cell markers within the same acquisition. In addition, CD20 expression levels were quantified on the basis of mean fluorescence intensity (MFI) values using the Kaluza software. Mature B cells have a well-defined CD20 expression and we considered this population as a positive control. To improve gating strategy, we set up our protocol and used Fluorescence Minus One (FMO) controls; the gating strategy and the calculation process are depicted in Figure S1. CS&T Beads (BD Biosciences, NJ, USA) with assigned values of molecules of equivalent soluble fluorochrome were used for longitudinal monitoring of instrument performance stability showing the low background of technical variance in MFI measurements during our study.

### 2.3. Data Analysis

Data analysis was performed using R 3.5.1 (R Foundation for Statistical Computing, Vienna, Austria). The difference between CD20 MFI at day 15 and at diagnosis will be further denoted as delta MFI CD20. Categorical variables were represented as absolute values (percent). Contingency tables were analyzed using Fisher’s exact test. The normality of the distribution was assessed using Shapiro–Wilk’s test, histogram visualization, and kurtosis and skewness assessment. All continuous variables assessed were non-normally distributed. Non-normally distributed variables were represented as median (quartile 1, quartile 3). Differences between two unpaired groups (either non-normally distributed or when assessing ordinal variables) were assessed using the Mann–Whitney–Wilcoxon rank sum test. Paired non-normally distributed variables were assessed using Mann–Whitney–Wilcoxon signed rank test. Patients were followed starting with diagnosis. If the event assessed did not occur at follow-up, the patient was right-censored on the day of follow-up. In the case of OS, the event was considered as death. In the case of RFS, the event was considered as the first relapse. Univariate survival analysis was assessed using a univariate Cox proportional hazards model. Multivariable survival analysis was assessed using a multivariable Cox proportional hazards model. Additionally, we used surv_cutpoint of the survminer package to determine an MFI cut-off point. A *p*-value under 0.05 was considered statistically significant.

## 3. Results

Between January 2017 and December 2020, 114 patients, 60.5% male and 39.5% female, were diagnosed with B-cell ALL, with a median follow-up of 2.7 years (range 1.6–4.2). A total of 69/114 patients were under 6 years old at diagnosis (median 5 with Q1, Q3 of (3, 9) and a range between 0–17 years), and we found a similar ratio of urban/rural provenance. 

The majority of patients (78.1%) were diagnosed with common ALL subtype, the rest having a pre-B (19.3%) or pro-B (2.6%) phenotype, in accordance with the criteria of the European Group for Immunologic Classification of Leukemias [16]. L1 FAB type was predominant with almost 94% of cases and 6% being accountable for the remaining cases.

An initial complete blood count revealed 77 patients (67.6%) with less than 20,000 leukocytes/μL, 22 patients (19.3%) with 20,000–50,000 leukocytes/μL and 15 patients (13.1%) with over 50,000 leukocytes/μL.

Successful cytogenetic analysis was performed on 83 patients (72.8%): 34 (29.8%) presented karyotype without anomalies, 21 (18.4%) hyperdiploidy and only 1 (0.9%) hypodiploidy. All patients were tested for the molecular anomalies: *TCF3::PBX1*-t(1;19)(q23;p13), *KMT2A::AFF1*-t(4;11)(q21;q23), *BCR::ABL* p190-t(9;22)(q34;q11), *BCR::ABL* p210-t(9;22)(q34;q11), *ETV6::RUNX1*-t(12;21)(p13;q22), *STIL::TAL1*-del(1)(p32;p32). The molecular biology results were: 37 (32.5%) of the testing positive for a fusion gene: 27 (23.7%) positive for *ETV6::RUNX1*, 7 (6.1%) for *BCR::ABL 1* and 2 (1.8%) for *TCF3::PBX1*.

Regarding treatment protocols, the patients were stratified in risk groups [13] and treated according to two sequential ALL BFM trial regimens [14,15]; as such, 59 (51.8%) patients received the ALL-IC BFM 2009 protocol while the AIEOP-BFM 2017 protocol was used for the remaining patients.

The first treatment evaluation was prednisone response, which was defined as the number of blasts in the peripheral blood sample per microliter at day 8 after the 7 day prednisone pre-phase with 96 patients (84.2%) having a good prednisone response (PGR < 1000 blasts/μL).

The second evaluation by FCM-MRD of bone marrow sample on day 15 revealed: MRD < 0.1% in 28 cases (24.6%), MRD ≥ 0.1–10% in 60 cases (52.6%), and MRD > 10% for 26 patients (22.8%) (Table 1).

Risk stratification was performed according to ALL-IC protocol criteria with 50 patients (43.9%) being assigned to the standard-risk group (SRG), 30 patients (26.3%) to the intermediate-risk group (IRG) and 34 patients (29.8%) to the high-risk group (HRG). 

**Figure 1:**
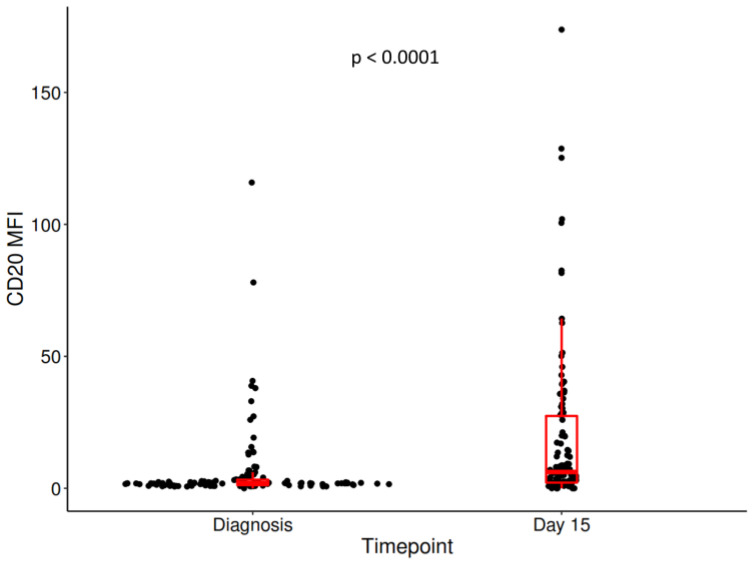
The difference in CD20 MFI between diagnosis and day 15. Centrality and dispersion measures were represented in the form of box and whiskers.

We observed an increase in the MFI of CD20 between diagnosis—1.9 (1.2–3.26) and day 15: 6.17 (2.14–27.4), (*p* < 0.0001). A significantly increased CD20 percentage at day 15—33.10% (5.24,76.33) compared with diagnosis—1.34 (0.35, 9.34) (*p* < 0.0001) was identified. The difference between CD20 MFI at day 15 and at diagnosis will be denoted as delta MFI CD20: 2.66 (range, 0.27–17.25) (Figure 1).

**Figure 2:**
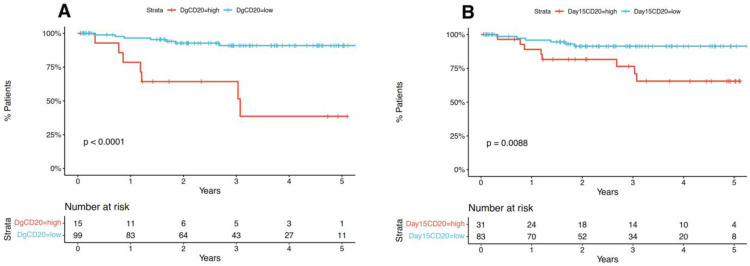
MFI CD20 diagnosis, day 15 and delta CD20 impact on RFS. (**A**,**B**) Survival was represented in the form of Kaplan-Meier plots and an accompanying table showing the number of patients at risk.

OS was measured from the date of diagnosis to the date of death from any cause. RFS was measured from the date of diagnosis to the date of recurrence from any cause. We established a cut-off for OS regarding MFI CD20 at diagnosis (>8.08), day 15 (>28.65) and delta CD20 (>19.18); and a cut-off for RFS regarding MFI CD20 at diagnosis (>8.08), day 15 (>19.99) and delta CD20 (>15.17). Using these cut-off points, we assessed if the high and low levels of CD20 MFI are associated with RFS and OS and observed that both diagnosis and day 15 CD20 MFI, but not delta CD20 MFI had an impact on RFS (Figure 2 and Figure S2) and OS (Figures S3–S5).

**Figure 3:**
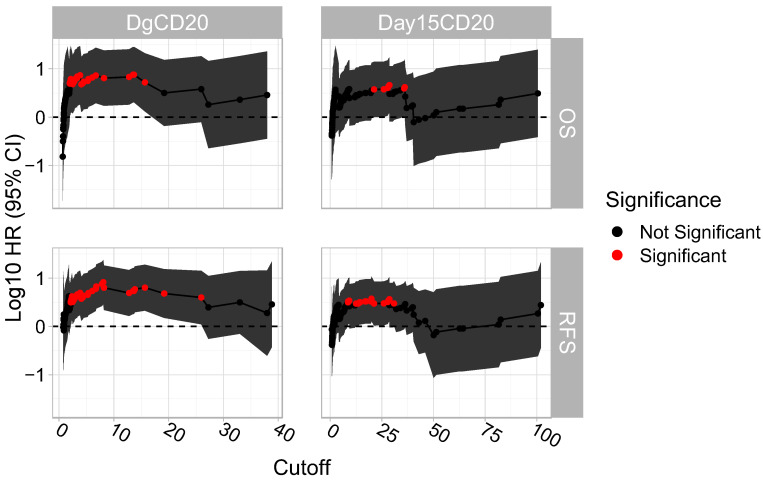
“Sliding window” approach on determining the succession of cut-off points that are significantly associated with RFS and OS. Each possible cut-off point was assessed and their respective log10 HR (95% CI) were plotted.

We then assessed the association between each unique possible cut-off of diagnosis CD20 and of day 15 CD20 with RFS and OS. We observed that for diagnosis CD20 there was a succession of cut-off points, including 8.08 which was significantly associated with both RFS and OS. A similar association was observed in the case of day 15 CD20, but the number of succeeding cut-off points and the association were lower (Figure 3).

We further assessed which variables were associated with RFS and OS. Of note, *ETV6::RUNX1* was also associated with a positive prognosis (RFS log-rank *p*-value = 0.02; RFS log-rank *p*-value = 0.06); however, due to the fact that no *ETV6::RUNX1* patients had an RFS or OS event, we could not use *ETV6::RUNX1* in the univariate Cox proportional hazards model (Table 2).

In addition, we determined which of the variables observed to be predictive of RFS and OS were associated with the groups generated using the CD20 MFI cut-offs.

Further, we assessed if there was an association between diagnosis and day 15 CD20 and other variables with RFS and OS. Most notably we observed that high CD20 MFI at diagnosis as well as at day 15 were associated with PPR (Table 3).

Further, we assessed if the association between diagnosis and day 15 CD20 was independently associated with RFS and OS when adjusting one at a time for the variables observed to be associated with RFS (Table 4) and OS (Table 5). In this case, diagnosis CD20 remained associated with both RFS and OS when adjusting for any relevant of the variables.

## 4. Discussion

In our study, patients were diagnosed with B-ALL, according to the BFM type protocol, including risk stratification criteria, demographic, morphologic, immunophenotypic, cytogenetic, and treatment response characteristics similar to reports from large BFM study groups. The male to female ratio of the patients in our study was analogous to other similar studies [17,18].

CD20 is an important surface antigen of B cells, commonly used as a marker in the diagnosis and monitoring of B-ALL. Specifically, CD20 is a 33–37 kDa non-glycosylated phosphoprotein expressed on the surface of normal and malignant B-lymphocytes and its expression starts from early development until it is lost with terminal plasma cell differentiation [19]. The biological function of CD20 is not fully understood, but it localizes with CD40, MHC class II and the B-cell receptor antigen (BCR) and has been suggested to be involved in B-cell receptor activation and proliferation, and enhancement of calcium signaling [20,21]. Several studies have shown that about 30–50% of ALL patients express CD20 on at least of 20% of leukemic blasts [10,11,12]; however, there is a paucity of data regarding CD20 expression and its impact on the outcome of B-ALL pediatric patients. The majority of our patients were under 6 years old, 69 (60.5%). Current studies show higher CD20 expression in children with B-ALL under 10 years old compared to those over 10 years old. This finding is relevant due to the prognostic value of CD20 expression and the use of rituximab base treatment in B-ALL pediatric patients. Borowitz et al. carried out the first large investigation into CD20 expression in children and young adults and determined a worse event-free survival (EFS) with a higher fluorescence intensity of CD20, whereas Jeha et al. identified that CD20 expression conferred a slightly favorable prognosis (5 year EFS rate of 84% ± 2.9% versus 78% ± 3.1%, *p* = 0.08) [22,23].

Watt et al. showed a phenotypic modulation that occurred in leukemic cells during induction treatment resulting in an up-regulation of CD20, a finding also reported by other authors [8,10,11,12,24]. The addition of anti-CD20 agents into chemotherapy regimens has improved the outcome for some non-Hodgkin lymphoma (NHL). Anti-CD20-directed immunotherapy, like rituximab, can significantly improve the outcome in B-cell precursor ALL in this population. In mature B-lineage (Burkitt-type) ALL, where high-intensity CD20 expression is a universal feature, the incorporation of rituximab into hyper-CVAD has significantly improved outcomes; direct mechanisms of action of Rituximab, which induces complement-dependent cytolysis, antibody-dependent cell-mediated cytotoxicity, and apoptosis are likely to play a role [11,25]. 

Our study established a cut-off for OS and RFS regarding MFI CD20 at diagnosis and day 15 and we observed that some traditional risk factors including age, leukocytes, prednisone response, MRD at day 15, together with MFI CD20 at diagnosis and at day 15, were associated with a negative impact on OS and RFS. Radu et al. evaluated all those important risk factors finding similar impacts regarding survival rates [26].

The differences in CD20 expression between days (deltaMFI-CD20 high) did not impact the OS and RFS. In the multivariable model, diagnosis MFI CD20 >8.08 seems to be more important than day 15 MFI CD20 in predicting both RFS and OS.

Our results are similar to those reported by the Pediatric Oncology Group (POG), where in among the 1231 patients 1 to 21.9 years old with newly diagnosed B-cell precursor ALL, CD20 expression was associated with an inferior treatment outcome. In addition, in a study on adults with de novo B-ALL, Thomas et al. described that CD20 positivity was associated with lower 3 year rates of complete remission duration (CRD; 20% vs. 55%, *p* < 0.001) and overall survival (OS; 27% vs. 40%, *p* = 0.03) [21,27]. 

One limitation of our study is the rather small number of patients included. In addition, a future potential improvement of our study protocol could represent the inclusion of an isotype control that would provide a negative control showing that there was weak or incomplete blocking of B cells expressing CD20, as shown by Maecker et al. [28]. Additional prospective studies are needed to determine the most favorable approach for anti-CD20 therapy in B-ALL pediatric patients with CD20 positive at diagnosis and possibly in those with CD20 negative based on up-regulation of CD20 expression. However, the findings of this study offer new, potentially useful information for pediatric patients with B-ALL.

## Figures and Tables

**Table 1:** General characteristics of the cohort.

Category	Variable	Value (Percentage)
Gender	Female	45 (39.5%)
	Male	69 (60.5%)
Region	Urban	56 (49.1%)
	Rural	58 (50.9%)
Age at diagnosis	Over 6 years	45 (39.5%)
	Under 6 years	69 (60.5%)
Leukocytes/µL	[880–3860]	22 (19.3%)
	(3860–10,000]	28 (24.6%)
	(10,000–20,000]	27 (23.7%)
	(20,000–50,000]	22 (19.3%)
	(50,000–100,000]	8 (7%)
	Over 100,000	7 (6.1%)
Peripheral blasts (percentage)	Median (Q1, Q3)	54 (10, 78)
Hemoglobin (g/dL)	Under 5	15 (13.2%)
	[5–7)	25 (21.9%)
	[7–10)	53 (46.5%)
	[10–13)	17 (14.9%)
	Over 13	4 (3.5%)
Platelets/uL	Under 10,000	7 (6.1%)
	[10,000–20,000)	10 (8.8%)
	[20,000–50,000)	42 (36.8%)
	[50,000–100,000)	28 (24.6%)
	[100,000–350,000)	27 (23.7%)
FAB	L1	107 (93.9%)
	L2	7 (6.1%)
Immunophenotype	B common	89 (78.1%)
	preB	22 (19.3%)
	proB	3 (2.6%)
Cytogenetics	No metaphases	31 (27.2%)
	Hyperdiploid	21 (18.4%)
	Hypodiploid	1 (0.9%)
	Normal	34 (29.8%)
	Other	27 (23.7%)
Molecular biology	No molecular alterations	77 (67.5%)
	*ETV6::RUNX1*	27 (23.7%)
	*BCR::ABL*	7 (6.1%)
	*TCF3::PBX1*	2 (1.8%)
	FLT3-ITD	1 (0.9%)
Prednisone response	PGR	96 (84.2%)
	PPR	18 (15.8%)
Risk group	SRG	50 (43.9%)
	IRG	30 (26.3%)
	HRG	34 (29.8%)
Protocol	ALL-IC-BFM 2009	59 (51.8%)
	AIEOP-BFM 2017	55 (48.2%)
MRD day 15	MRD < 0.1%	28 (24.6%)
	MRD ≥ 0.1–10%	60 (52.6%)
	MRD > 10%	26 (22.8%)
MFI CD20 diagnosis	Median (Q1, Q3)	1.90 (1.20, 3.26)
Percentage CD20+ blasts diagnosis	Median (Q1, Q3)	1.34 (0.35, 9.34)
MFI CD20 day 15	Median (Q1, Q3)	6.17 (2.14, 27.4)
Percent CD20+ blasts day 15	Median (Q1, Q3)	33.10 (5.24, 76.33)
Delta MFI CD20	Median (Q1, Q3)	2.66 (0.27, 17.25)

Continuous variables used in the downstream analysis (age at diagnosis, leukocytes, hemoglobin, platelets, MRD day 15) were converted into ranks (starting from the lowest numerical values) based on the cut-offs presented in the current table. The same was performed for the ordinal variable used (risk group) with SRG receiving a rank of 1, IRG a rank of 2 and HRG a rank of 3. FAB = The French-American-British, L1 = small uniform cells, L2 = large varied cells, *ETV6::RUNX1* = t(12;21) chromosome translocation, *BCR::ABL* = ABL gene from chromosome 9 joins to the BCR gene on chromosome 22, to form the *BCR::ABL* fusion gene, *TCF3::PBX1* = t(1;19) chromosomal translocation, FLT3-ITD = fms-related tyrosine kinase-3 gene internal tandem duplication mutation, PGR = prednisone good responder, PPR = prednisone poor responder, SRG standard risk group, IRG = intermediate risk group, HRG = high risk group, ALL-BFM = acute lymphoblastic leukemia-Berlin-Frankfurt-Münster consortium, AIEOP-BFM = Italian Association of Pediatric Hematology and Oncology-Berlin-Frankfurt-Münster consortium, MRD = minimal residual disease, MFI = mean fluorescence intensity, Delta MFI = the difference between CD20 MFI at day 15 and at diagnosis.

**Table 2:** Variables associated with RFS and OS.

Variable	Relapse Free Survival		Overall Survival	
	HR (95% CI)	*p*-Value	HR (95% CI)	*p*-Value
Male gender	0.85 (0.3, 2.46)	0.77	0.5 (0.13, 1.87)	0.30
Urban region	0.64 (0.22, 1.85)	0.41	0.7 (0.19, 2.6)	0.59
Over 6 years at diagnosis	4.25 (1.33, 13.56)	0.01	13.67 (1.71, 109.42)	0.01
Leukocytes	1.6 (1.11, 2.31)	0.01	1.9 (1.2, 3.02)	<0.01
Hemoglobin	1.12 (0.66, 1.89)	0.68	1.05 (0.54, 2.04)	0.89
Platelets	0.96 (0.59, 1.56)	0.87	1.23 (0.64, 2.36)	0.53
L2 FAB	2.76 (0.62, 12.41)	0.18	1.91 (0.24, 15.28)	0.54
PPR	5.76 (1.98, 16.72)	<0.01	5.84 (1.56, 21.85)	<0.01
Risk group	3.12 (1.41, 6.92)	<0.01	2.37 (0.96, 5.83)	0.06
ALL-IC-BFM 2017 protocol	0.75 (0.24, 2.31)	0.61	1.12 (0.28, 4.54)	0.87
MRD day 15	2.81 (1.2, 6.55)	0.02	2.22 (0.8, 6.19)	0.13
MFI CD20 diagnosis >8.08	8.23 (2.87, 23.61)	<0.0001	9.89 (2.64, 37.07)	<0.001
MFI CD20 day 15 >19.99	3.74 (1.3, 10.79)	0.01	3.55 (0.95, 13.26)	0.06
MFI deltaCD20 day 15 >28.65	3.65 (1.28, 10.42)	0.02	4.59 (1.23, 17.19)	0.02

Univariate Cox proportional hazards model.

**Table 3:** Association between the groups formed by using the CD20 MFI cut-offs and variables observed to be predictive of RFS and OS.

Variable		Diagnosis CD20 MFI			Day 15 CD20 MFI			Day 15 CD20 MFI		
		Over 8.08	Under 8.08	*p*-Value	Over 19.99	Under 19.99	*p*-Value	Over 28.65	Under 28.65	*p*-Value
		*n* = 15	*n* = 99		*n* = 31	*n* = 83		*n* = 26	*n* = 88	
Age at diagnosis	>6 years	6 (40%)	39 (39.4%)	1	13 (41.9%)	32 (38.6%)	0.83	11 (42.3%)	34 (38.6%)	0.82
	<6 years	9 (60%)	60 (60.6%)		18 (58.1%)	51 (61.4%)		15 (57.7%)	54 (61.4%)	
Leukocytes		2 (1, 3)	2 (1, 3)	0.23	2 (1, 3)	2 (1, 3)	0.55	2 (1, 3)	2 (1, 3)	0.54
Prednisone response	PGR	10 (66.7%)	86 (86.9%)	0.06	23 (74.2%)	73 (88%)	0.09	18 (69.2%)	78 (88.6%)	0.03
	PPR	5 (33.3%)	13 (13.1%)		8 (25.8%)	10 (12%)		8 (30.8%)	10 (11.4%)	
Risk group		2 (1, 3)	2 (1, 3)	0.35	2 (1, 3)	2 (1, 3)	0.07	2 (1, 3)	2 (1, 3)	0.15
MRD day 15		2 (2, 3)	2 (2, 2)	0.20	2 (2, 3)	2 (1, 2)	0.05	2 (2, 3)	2 (2, 2)	0.16
*ETV6::RUNX1*		0 (0%)	27 (27.3%)	0.02	5 (16.1%)	22 (26.5%)	0.33	3 (11.5%)	24 (27.3%)	0.12

**Table 4:** Multivariable Cox regression model analysis of RFS.

	Diagnosis CD20 MFI >8.08		Day 15 CD20 MFI >19.99		Day 15 CD20 MFI >28.65	
Variable	HR (95% CI)	*p*-Value	HR (95% CI)	*p*-Value	HR (95% CI)	*p*-Value
Over 6 years at diagnosis	15.25 (4.47, 52.05)	<0.0001	4.33 (1.49, 12.59)	<0.01	4.19 (1.46, 12.06)	<0.01
Leukocytes	7.23 (2.5, 20.93)	<0.001	3.93 (1.35, 11.39)	0.01	3.7 (1.29, 10.6)	0.01
PPR	6.08 (2.02, 18.33)	<0.01	3.18 (1.09, 9.28)	0.03	2.84 (0.97, 8.34)	0.06
Risk group	6.92 (2.37, 20.16)	<0.001	2.98 (1.02, 8.68)	0.05	2.86 (0.99, 8.25)	0.05
MRD day 15	6.33 (2.13, 18.8)	<0.001	2.82 (0.95, 8.42)	0.06	2.77 (0.94, 8.18)	0.07
*ETV6::RUNX1*	5.68 (1.98, 16.29)	<0.01	3.45 (1.20, 9.96)	0.02	3.11 (1.09, 8.87)	0.03

**Table 5:** Multivariable Cox regression model analysis of OS.

	Diagnosis CD20 MFI >8.08		Day 15 CD20 MFI >19.99		Day 15 CD20 MFI >28.65	
Variable	HR (95% CI)	*p*-Value	HR (95% CI)	*p*-Value	HR (95% CI)	*p*-Value
Over 6 years at diagnosis	21.44 (4.49, 102.44)	<0.001	4.38 (1.16, 16.55)	0.03	5.73 (1.5, 21.92)	0.01
Leukocytes	8.64 (2.26, 33.07)	<0.01	4.16 (1.09, 15.91)	0.04	5.03 (1.32, 19.18)	0.02
PPR	7.49 (1.88, 29.76)	<0.01	3.02 (0.8, 11.47)	0.10	3.62 (0.94, 13.99)	0.06
Risk group	8.79 (2.31, 33.53)	<0.01	2.94 (0.77, 11.13)	0.11	3.75 (0.98, 14.32)	0.05
MRD day 15	8.41 (2.16, 32.84)	<0.01	2.9 (0.74, 11.31)	0.12	3.83 (0.98, 14.94)	0.05
*ETV6::RUNX1*	6.80 (1.81, 25.49)	<0.01	3.28 (0.88, 12.27)	0.08	3.90 (1.04, 14.59)	0.04

## Data Availability

The data presented in this study are available on reasonable request from the corresponding authors.

## Supplementary Materials

The following supporting information can be downloaded at: https://www.mdpi.com/article/10.3390/jcm12041451/s1, Figure S1: Flow cytometric immunophenotyping in a pediatric patient with B ALL. The FMO control is performed by staining the cells of interest with all fluorochromes from our panel except PB (CD20). CD20 expression levels were quantified on the basis of mean fluorescence intensity (MFI) values using the Kaluza software. Figure S2: Kaplan–Meier curves representing the effect of the selected cut-off points of delta CD20 MFI on RFS, Figure S3: Kaplan–Meier curves representing the effect of the selected cut-off points of diagnosis CD20 MFI on OS, Figure S4: Kaplan–Meier curves representing the effect of the selected cut-off points of day 15 CD20 MFI on OS, Figure S5: Kaplan–Meier curves representing the effect of the selected cut-off points of delta CD20 MFI on OS.
