# Supplementary material for: The Significance of CD20 Intensity Variance in Pediatric Patients with B-Cell Precursor Acute Lymphoblastic Leukemia
Source: J Clin Med. 2023 Feb 11;12(4):1451. doi: 10.3390/jcm12041451 (PMC9961970; doi:10.3390/jcm12041451)
Supplement: Supplementary file 1 [file jcm-12-01451-s001.zip › jcm-2139944-supplementary.pdf]

Journal of Clinical Medicine

Article

The Significance of CD20 Intensity Variance in Pediatric Pa-tients with B-cell Precursor Acute Lymphoblastic Leukemia

Supplementary

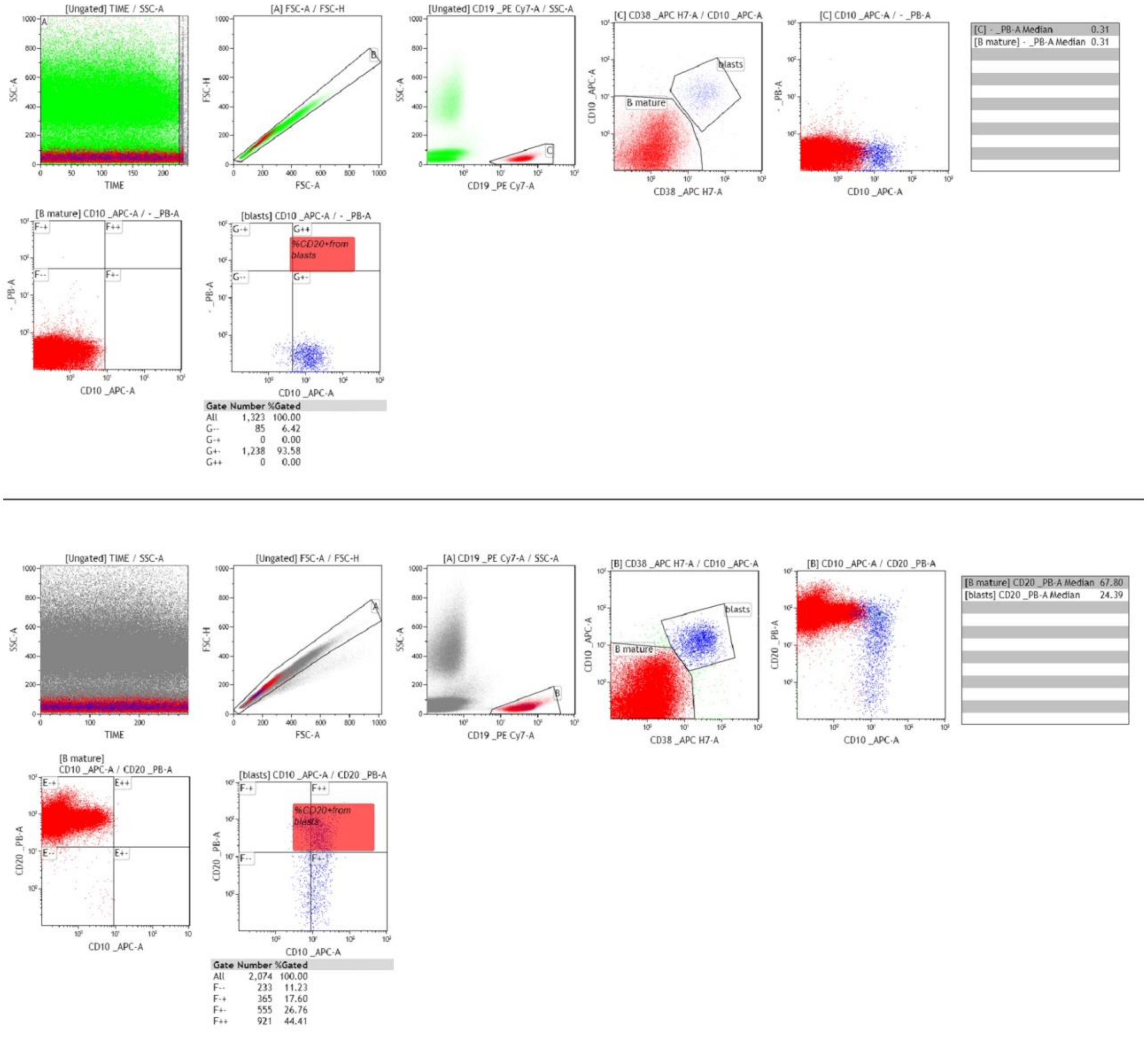

Figure S1: Flow cytometric immunophenotyping in a pediatric patient with B ALL. The FMO control isperformed by staining the cells of interest with all fluorochromes from our panel except PB (CD20). CD20 expression levels were quantified on the basis of mean fluorescence intensity (MFI) values using the Kaluza software.

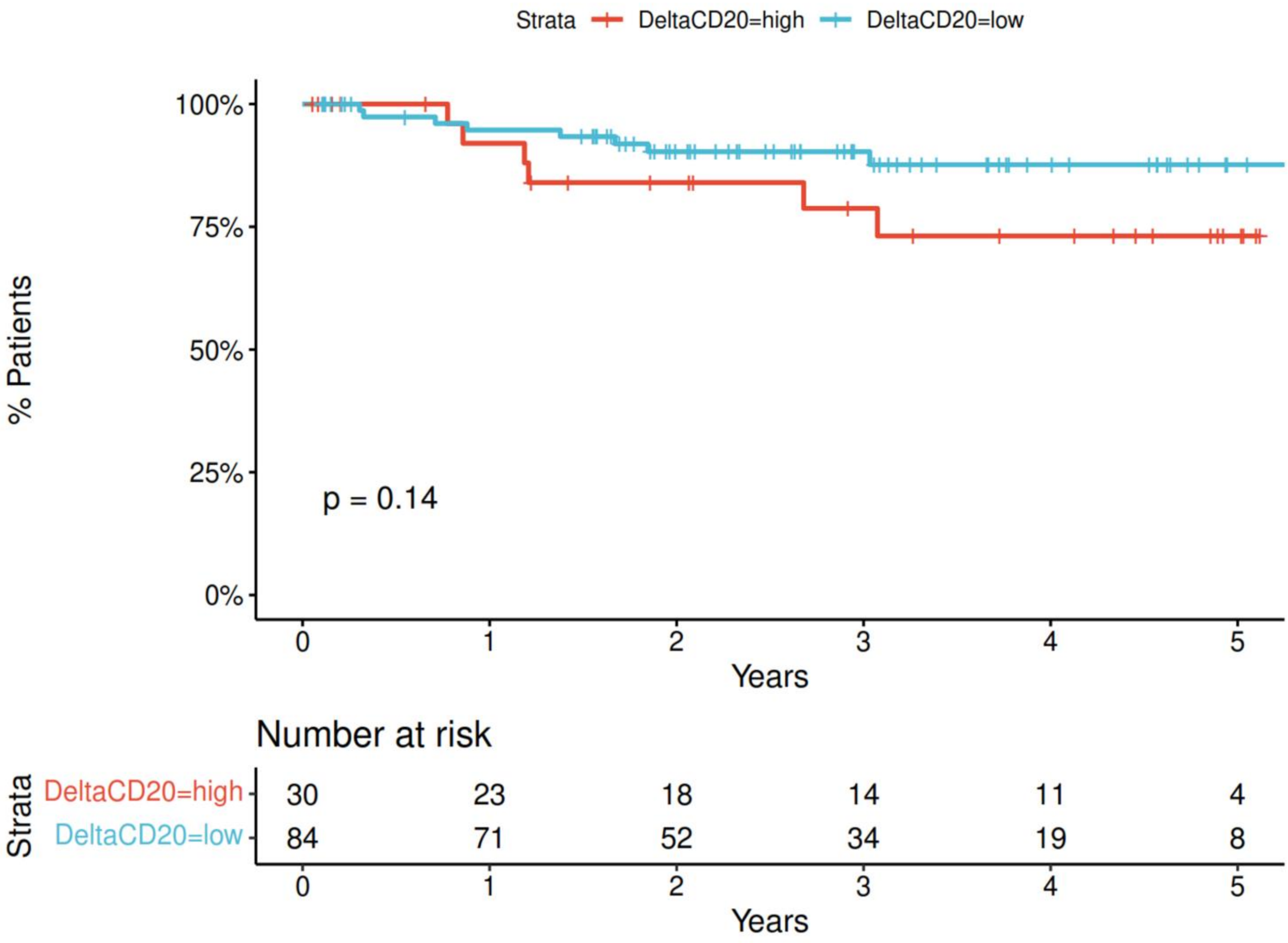

Figure S2: Kaplan-Meier curves representing the effect of the selected cutoff points of delta CD20 MFI on RFS.

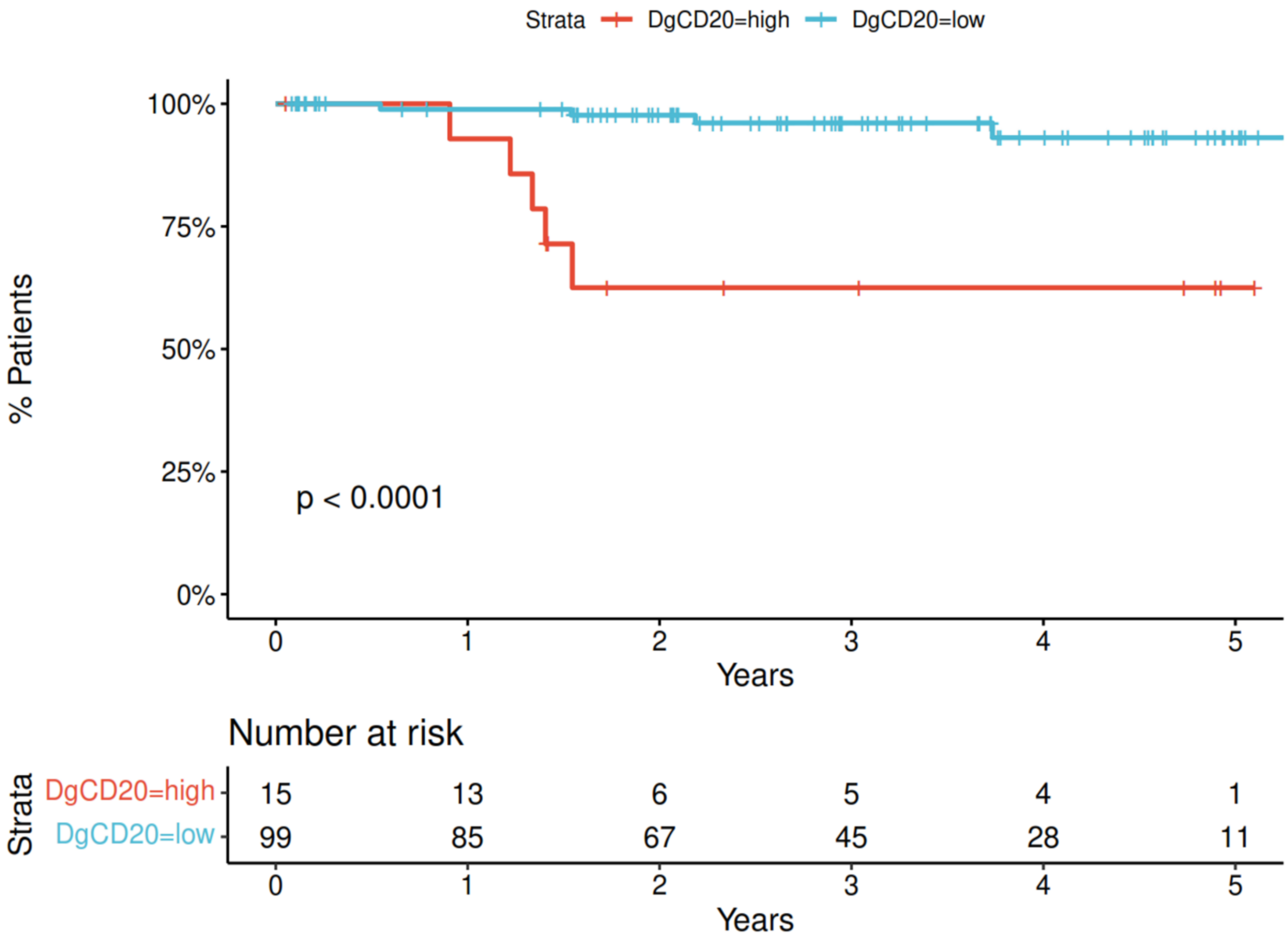

Figure S3: Kaplan-Meier curves representing the effect of the selected cutoff points of diagnosis CD20 MFI on OS.

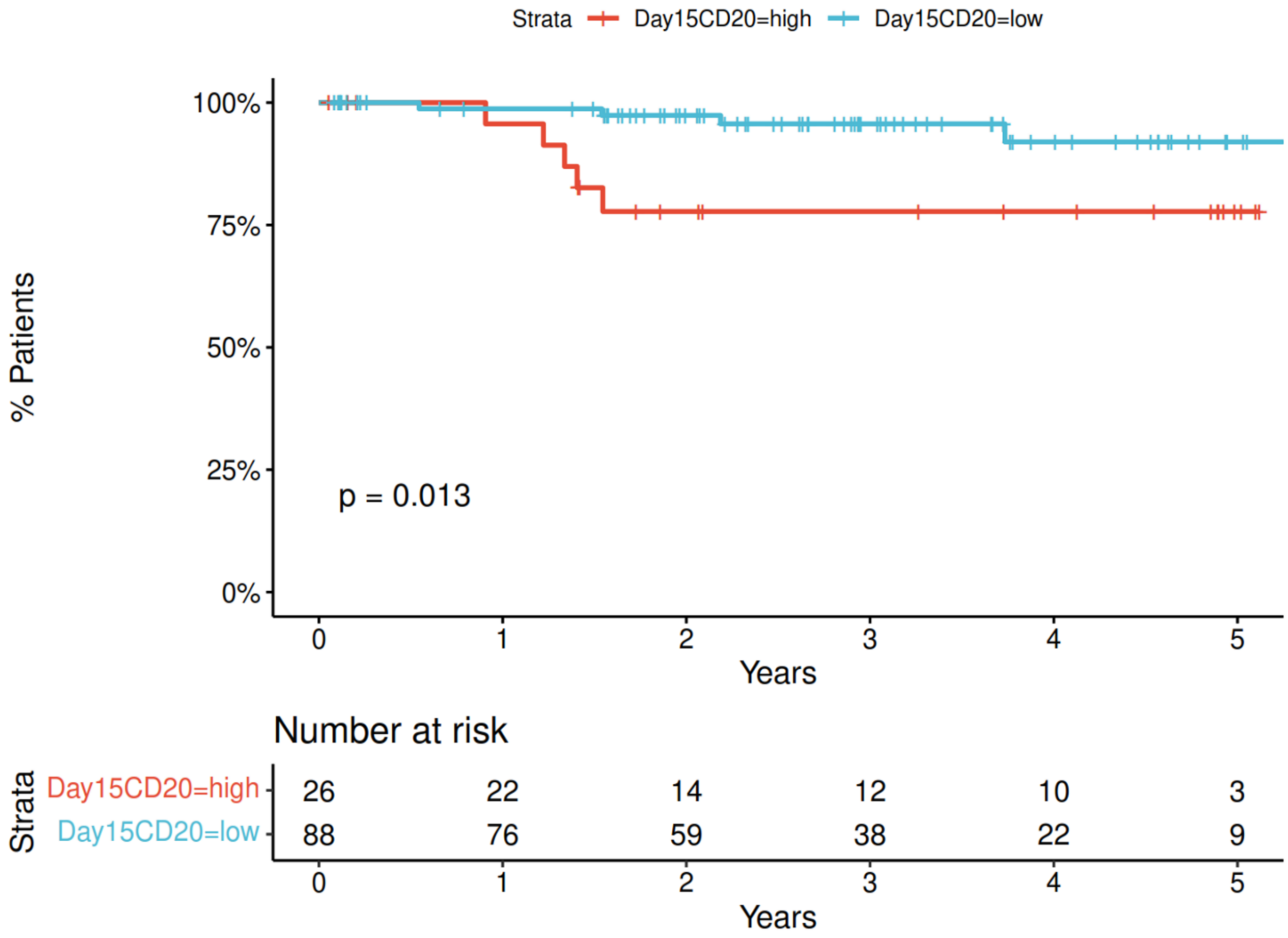

Figure S4. Kaplan-Meier curves Figure S4: Kaplan-Meier curves representing the effect of the selected cutoff points of day 15 CD20 MFI on OS.

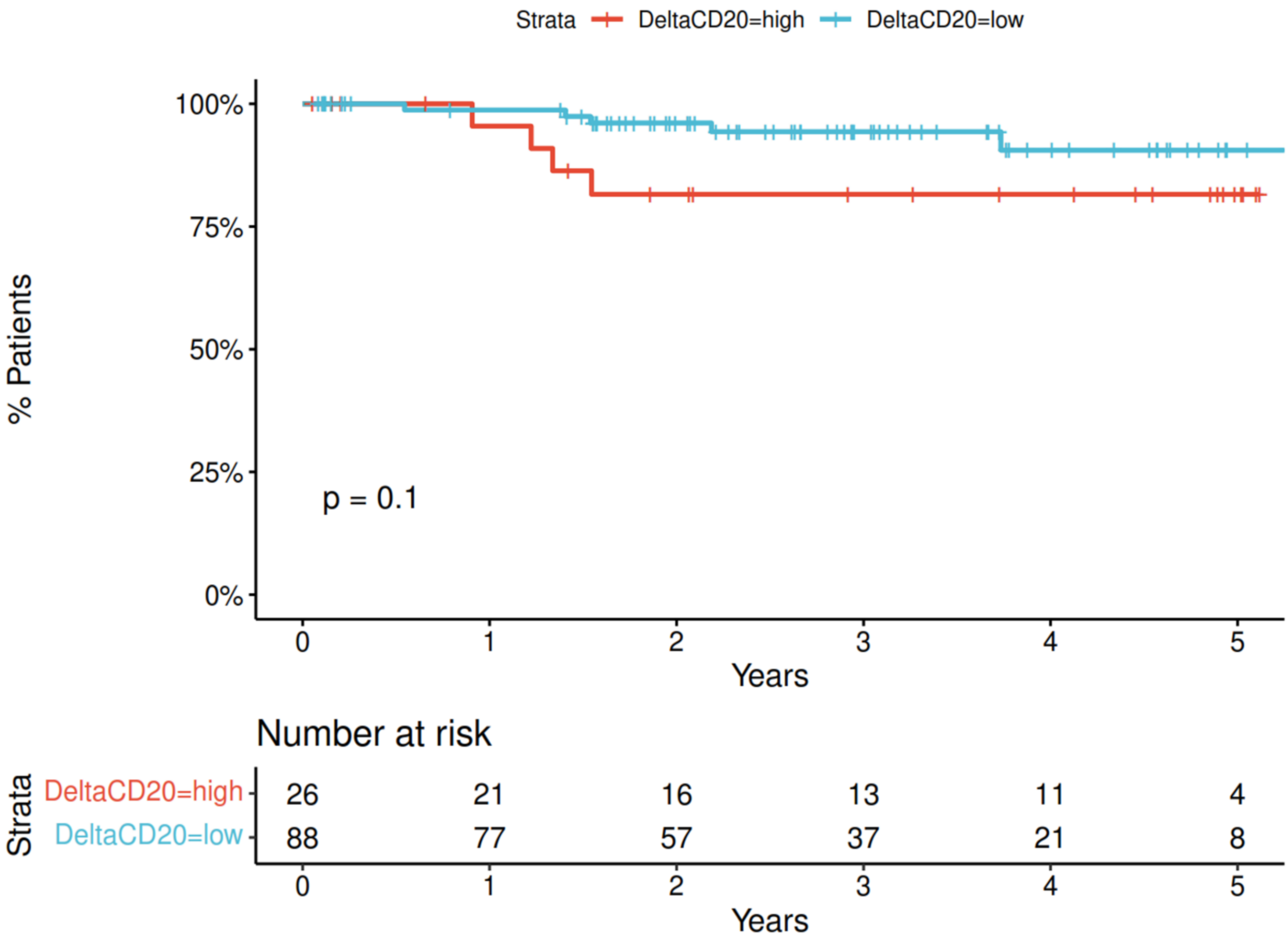

Figure S5: Kaplan-Meier curves representing the effect of the selected cutoff points of delta CD20 MFI on OS.
